# Self-Reported Health Inequalities among Older Adults in Saudi Arabia

**DOI:** 10.3390/healthcare12010072

**Published:** 2023-12-28

**Authors:** Mohammed Khaled Al-Hanawi

**Affiliations:** 1 Department of Health Services and Hospitals Administration, Faculty of Economics and Administration, King Abdulaziz University, Jeddah 21589, Saudi Arabia; mkalhanawi@kau.edu.sa; 2 Health Economics Research Group, King Abdulaziz University, Jeddah 21589, Saudi Arabia

**Keywords:** health inequalities, older adults, public health, Saudi Arabia, self-reported health, socio-economic status

## Abstract

Considering the rising life expectancy, the growing population of older adults poses challenges in providing adequate healthcare services. Self-reported health is an important indicator of overall health, predicting morbidity and mortality. This study investigated self-reported health inequalities among older adults in Saudi Arabia and the underlying factors contributing to establishing such inequalities. The study utilized data from the 2018 Saudi Family Health Survey, focusing on 2023 respondents aged ≥60 years with complete data. Univariate, bivariate, and multivariate logistic regression analyses were employed to explore socio-economic factors linked to health inequalities. Additionally, concentration curves and indices were used to assess the magnitude of health inequalities among older adults. The findings indicate a higher prevalence of self-reported poor health among respondents aged ≥70 years and those with chronic diseases. Age, education, income level, marital status, and insurance coverage were other factors significantly linked to reporting poor health. Inequality analysis revealed a concentration of poor health among less educated individuals (concentration index = −0.261, *p* < 0.01). Both income- and education-based indices highlighted a concentration of poor health among men with lower income and education levels. Addressing healthcare inequalities among older adults requires targeted policy efforts, focusing on those aged ≥70, unmarried individuals, those without insurance coverage, those with chronic illnesses, and those with lower education levels. Targeted interventions for these groups can address their unique healthcare needs and promote equitable health outcomes.

## 1. Introduction

In recent decades, global population aging has accelerated significantly, with people now living longer than before. Projections suggest that from 2015 to 2050, the percentage of individuals aged over 60 years will nearly double, increasing from 12% to 22% of the world’s population [1]. While the shift toward an aging population reflects progress in healthcare and societal development, it also poses challenges, particularly in ensuring healthcare access for older adults. The health of the population tends to decline gradually from early adulthood to retirement age [2]. Accumulated hazardous exposures and lifestyle changes influenced by societal advancements contribute to this phenomenon [3]. The older population faces higher health risk factors but encounters systematic challenges in healthcare access compared to younger age groups [4,5]. With escalating healthcare spending, the health challenges of older adults pose additional complexities for the healthcare system.

Health inequalities encompass variations and disparities in health outcomes among a population [6]. Such disparities may arise when society fails to mitigate conditions for disadvantaged groups. Self-reported health is an important indicator of overall health, predicting morbidity and mortality [7]. Research indicates that lower socio-economic groups tend to self-report being in poor health more frequently than other demographic groups [8,9]. Public health policies must therefore aim to address these inequalities, particularly to improve the health of vulnerable groups [10]. Besides lower socio-economic status, older adults are also considered to be a vulnerable group due to the natural decline in health and physical strength associated with aging [11]. The continuous rise in the older population has an impact on various aspects of healthcare, including staffing, care costs, program availability, and service provision [12]. Older individuals often face challenges in accessing necessary preventive, diagnostic, treatment, and management healthcare services [3,13]. From an economic perspective, population aging has a negative impact on economic growth due to increased pensions, per capita healthcare expenditures, and the indirect influence on national funding in other domains [14,15].

The impact of population aging is particularly significant in the Arab world, where the older population is projected to reach the highest proportion of the total population by 2050 [16]. In the Kingdom of Saudi Arabia (KSA), the trend of population aging is prominent, with the number of individuals aged ≥65 years projected to continue growing. By 2050, this age group is anticipated to comprise 18.4% of the country’s total population [14]. This is attributable to the KSA’s notable advancements in enhancing the health standards of its population compared to other countries in Western Asia [17]. Nevertheless, the public healthcare services in the KSA face the challenges of being overburdened and overcrowded [18]. As such, the country is likely to encounter additional difficulties arising from population aging [19].

Although the implications of the aging population are significant for the KSA, there is a scarcity of literature regarding the health status of older adults in the country. Existing research has primarily focused on the burden experienced by informal caregivers of older individuals [20], non-communicable diseases (NCDs) among the older population [19], and the perspectives of older individuals on health [14]. In the context of health inequalities in the KSA, existing literature has explored determinants through a systematic review [21], socio-economic inequalities in diabetes prevalence [22], gender-based health inequalities [23], and socio-economic variations in dental service utilization [24]. Nevertheless, these studies, besides their limited scope and lack of empirical techniques, do not specifically address health inequalities among older adults.

To fill this gap, this study examined self-reported health inequalities among older adults (aged ≥ 60 years) in the KSA, aiming to identify its existence and explore the underlying factors contributing to these inequalities. Data from the 2018 Saudi Family Health Survey (FHS) were utilized for this analysis. Employing univariate, bivariate, and multivariate logistic regression analyses, we aimed to identify socio-economic factors linked to self-reporting being in “poor” or “good” health among the older generation to determine the existence of health inequalities and the contributing factors. Additionally, concentration curves and indices were used to assess the magnitude of health inequalities among older adults participating in the survey.

This study focuses on Saudi Arabian individuals aged 60 years and above, thereby contributing to the existing literature in multiple ways. Firstly, it evaluates the applicability of international findings on older adults to the specific context of the KSA. Secondly, it employs empirical techniques to enhance and complement findings from previous studies lacking such investigative methods. Thirdly, in contrast to studies with narrower scopes, this research examines health inequalities across various socio-economic characteristics. Lastly, given the increasing trend of population aging in the country, this research adds knowledge about the overall health status of older adults, with implications for policymaking regarding their health outcomes. In general, this study offers insights that can inform the formulation of public policies and interventions geared toward improving healthcare services, with a particular focus on addressing the needs of older adults facing socio-economic disadvantages.

## 2. Materials and Methods

### 2.1. Data Source and Sample

This study used nationally representative secondary data obtained from the 2018 Saudi FHS conducted by the General Authority for Statistics (GaStat) [25]. The FHS is a field survey classified under the categories of education and health statistics, representing a collaboration between the GaStat, Ministry of Health, Saudi Health Council, academic institutes, and the private sector. Surveys were completed based on visits to 15,265 individuals who were randomly selected across all 13 administrative regions of the KSA using a two-stage sampling approach to obtain a representative sample of the entire population.

The survey contains questions related to geography, health status, health utilization, and chronic diseases, among other topics addressing family and health issues. Several health variables were considered in the present study, with a focus on the report of self-assessed health status. The present analysis was limited to data obtained only from respondents aged ≥ 60 years who provided complete information on all variables of interest, resulting in an analyzed sample of 2023 respondents.

### 2.2. Measurement Variables

In the FHS, the self-reported health (SRH) status was determined from the following question: “how do you classify your health in general, which includes both your physical and mental health?”. The responses were scored on a 5-point scale: “Very good”, “Good”, “Mediocre”, “Bad”, and “Very bad”. SRH status was used as the outcome variable for examining the socio-economic determinants and health inequalities among older adults in the KSA. To facilitate the bivariate analysis, the 5-point scale was converted to a binary outcome of 0 and 1, with 0 representing responses “Very good”, “Good” and “Mediocre” as good health, and 1 representing the responses “Bad” and “Very bad” as poor health [26,27,28].

Independent variables considered in the model were socio-economic and demographic characteristics such as age, gender, marital status, nationality, education level, monthly income, health insurance coverage, and the existence of chronic illness. Among these, income and education level served as the indicators of socio-economic status. Respondents were classified into two age categories: younger (60 to 69 years) coded 0 and older (≥70 years) coded 1. Gender (1 for man and 0 for woman), marital status (1 for married and 0 for unmarried, including never married, divorced, and widowed), nationality (1 for Saudi and 0 for non-Saudi), health insurance (1 covered and 0 not covered), and chronic illness status (1 present and 0 absent) were all captured as binary variables. Education level was divided into five groups: below primary school (reference), primary school, intermediate school, high school, and higher education. Monthly income (in Saudi Riyal (SR); 1 SR = USD 0.27) was grouped into eight categories: <3000 (reference category), 3000 to <5000, 5000 to <7000, 7000 to <10,000, 10,000 to <15,000, 15,000 to <20,000, 20,000 to <30,000, and ≥30,000.

### 2.3. Statistical Analysis

The analysis for this study was conducted in various steps. Initially, univariate analyses were performed to evaluate the relative frequencies of respondents for each characteristic. Bivariate analysis was performed for the cross-tabulation of the dependent variable (SRH) with the associated frequencies using a Chi-squared test. Multivariate logistic regression models were used to examine the independent associations of socio-economic factors with SRH controlling for age, gender, marital status, nationality, health insurance coverage, and chronic illness status. Socio-economic health inequalities at the national level and in each socio-economic group (according to income and education levels) were evaluated based on the construction of the concentration curve and the calculation of the concentration index according to the methodology of Wagstaff et al. [29].

The concentration index (ranging from −1 to +1) is calculated as twice the area between the concentration curve and the line of equality [30], whereby a positive index value indicates that a given health variable is disproportionately concentrated among the rich/highly educated and a negative index value indicates concentration among the poor/less educated. The concentration curve offers a visual representation of the degree of inequality in SRH by plotting the cumulative percentage of a health variable with respect to the cumulative share of that variable in the population (variables are ranked according to a socio-economic status indicator from the lowest to the highest) [30]. For example, with respect to income and education level, a curve above the line of equality (i.e., the 45-degree line) indicates that poor health status is concentrated among those with lower income/less education, whereas a concentration curve below the line of equality indicates that poor health status is concentrated among those with higher income/higher education. The further the concentration curve diverges from the line of equality, the greater the degree of inequality.

## 3. Results

### 3.1. Descriptive Statistics

The socio-demographic characteristics of the analyzed respondents are presented in Table 1. Among the total sample of 2023 respondents aged ≥60 years, 17.35% rated their health status as poor, while 82.65% rated their health status as good. Over half of the respondents were men, three-quarters were married, over half had an education level below primary school, approximately 21% of the respondents earned 15,000 SR and more, around three-quarters were covered by health insurance, and the majority (85.91%) were suffering from a chronic illness.

### 3.2. Bivariate Analysis

The results of the bivariate analysis between SRH and socio-economic characteristics are presented in Table 2. Poor health status was significantly associated with age (χ^2^ = 46.05, *p* < 0.01), which was mainly concentrated among the older respondents (aged ≥ 70 years; 23.61%) compared to the younger respondents (60–69 years; 12.14%). Gender (χ^2^ = 32.23, *p* < 0.01), marital status (χ^2^ = 51.17, *p* < 0.01), and educational attainment (χ^2^ = 78.72, *p* < 0.01) were significantly associated with reporting a poor health status. In particular, the frequency of reporting a poor health status was much lower (2.94%) among people with a higher education than among respondents with below primary school (23.43%) or primary school (11.73%) education. Moreover, reporting a poor health status was significantly associated with health insurance status (χ^2^ = 33.58, *p* < 0.01), which was mainly concentrated among the uninsured (20.37%) compared to the insured (9.46%). Similarly, reporting a poor health status was significantly associated with chronic illness status (χ^2^ = 35.79, *p* < 0.01), in which reporting a poor health status was more heavily concentrated among those suffering from chronic diseases (19.39%) than those not suffering from chronic diseases (4.91%).

The Wagstaff inequality concentration index values to quantify the level of inequalities in SRH across socio-economic categories (i.e., income and education) are presented in Table 3.

The overall education-based concentration index was −0.261, which was statistically significant at the 1% level, demonstrating a greater tendency to report poor health status among the less-educated respondents in the KSA. Gender also impacted both income-based and education-based concentration indices, with poor health status concentrated among men with lower income and lower education levels. Age was a significant factor negatively associated with education-based concentration indices, whereas the income-based concentration index for the younger group (60–69 years) was positive, indicating a greater concentration of poor health status among those in their 60s with a higher income level.

**Figure 1:**
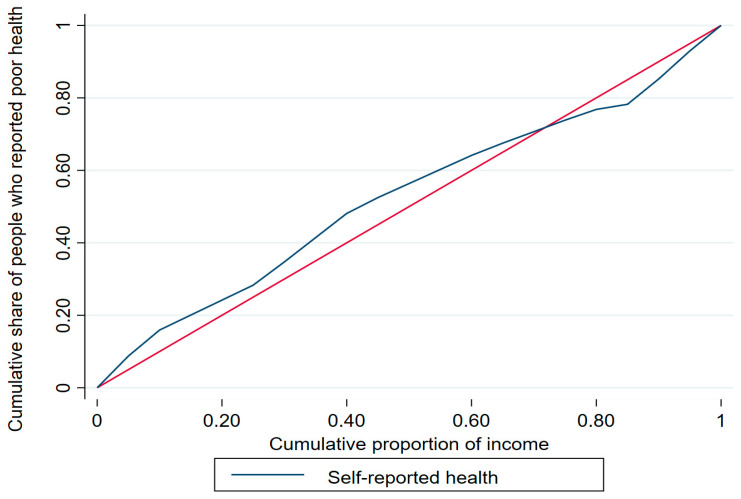
Income-based concentration curve.

**Figure 2:**
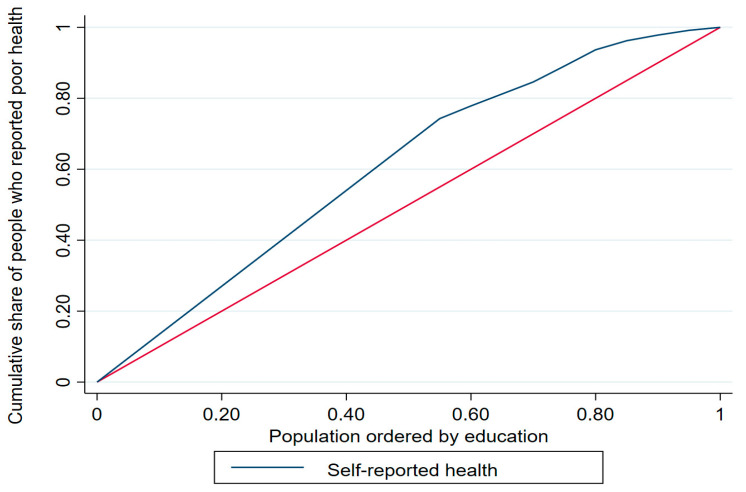
Education-based concentration curve.

The concentration curves for income and education are shown in Figure 1 and Figure 2, respectively. The income-based concentration curve for poor health status (blue) almost overlaps with the line of equality (red), suggesting no inequality, whereas the education-based concentration curve is above the line of equality, confirming the prevalence of poor health status among the less educated older adults in the KSA.

### 3.3. Regression Analysis

Multivariate logistic regression analysis was performed to consider the potential influence of other variables on the association between poor health status and socio-economic factors. Table 4 summarizes the results. Model 1 showed that the likelihood of reporting a poor health status was lower among all income groups (except those who earn 20,000 to 30,000 and ≥30,000 SR) when compared with the lower income group (earning below 3000 SR). For example, the odds ratio (OR) was 0.247 (95% confidence interval [CI]: 0.104–0.586; *p* ˂ 0.01) for people with a reported income level of 15,000 to <20,000 SR. Model 2 indicated the lower likelihood of reporting a poor health status among people with a higher education level (OR: 0.225, 95% CI: 0.078–0.645; *p* ˂ 0.01) when compared with less educated people (below the primary school level of education). The OR of education categories remained statistically significant in Model 3, along with a greater likelihood of reporting a poor health status among people aged ≥70 years (OR: 2.035, 95% CI: 1.547–2.675; *p* ˂ 0.01) when compared with that of the younger group aged 60–69 years.

In addition, married respondents had a lower likelihood of reporting a poor health status (OR: 0.566, 95% CI: 0.393–0.815; *p* ˂ 0.01) when compared with unmarried respondents, indicating that reporting a poor health status was almost 0.57 times lower for those who are married than for those who are unmarried. Moreover, the likelihood of reporting poor health status among those with health insurance coverage was lower when compared with that of those who are not insured (OR: 0.480, 95% CI: 0.335–0.687; *p* ˂ 0.01). Model 3 also revealed the higher likelihood of reporting poor health status among those with a chronic illness (OR: 4.089, 95% CI: 2.305–7.251; *p* ˂ 0.01) when compared with those not suffering from a chronic illness, suggesting that those with a chronic illness were 4.1 times more likely to report a poor health status when compared with those not suffering from a chronic illness.

## 4. Discussion

This study examined the self-reported health inequalities among older adults (≥60 years) in the KSA and explored the underlying factors driving the occurrence of this phenomenon. By utilizing a rich dataset from the 2018 Saudi FHS, which provides an advantage of national representativeness, this study offers a detailed analysis of health inequalities. To ensure that the results of this study would be reliable and robust, multiple techniques were employed, including descriptive analysis using percentages and frequencies, bivariate analysis using the Chi-square test, inequality analysis using the Wagstaff concentration index, and regression analysis using the logistic model. These methodologies strengthen the findings, thereby maintaining their policy relevance in informing interventions aimed at addressing health inequalities among the older population.

Age was a significant factor associated with self-reporting poor health, showing a higher prevalence among individuals aged ≥70 (23.61%) compared to those in the younger group (60–69 years). This finding aligns with the existing literature indicating an increasing prevalence of poor health with increasing age [28,31,32]. For instance, a study in Brazil found an association of age with mortality in older men, with each additional year increasing the risk of death by 5% [33]. In the KSA, the observed poor health among older adults is consistent with the overall high prevalence of NCDs in the adult population [19,34]. Factors such as limited physical activity, unhealthy habits, financial constraints, and limited access to specialized care contribute to the hindered health status of older individuals. Additionally, research conducted by Haseen et al. [32] and Molarius et al. [35] emphasizes that the natural aging process leads to functional limitations and a weakened immune system, both of which negatively impact the health status of older individuals. Therefore, it is essential to educate the population on practical health measures to alleviate the impact of the aging process.

Significant associations were found between reporting a poor health status and marital status. Marriage was associated with a lower likelihood of self-reporting a poor health status compared to unmarried individuals, indicating an approximately 0.57 times lower likelihood of self-reporting a poor health status for married individuals. Unmarried older individuals, who live alone, often experience lower social participation, accompanied by increased feelings of loneliness given the lack of emotional and practical support at home [36]. This finding aligns with a previous study showing that family interactions were viewed as crucial by older individuals in the KSA [14]. The absence of spousal support may contribute to poor dietary habits and an undisciplined lifestyle, ultimately leading to poor health among the older population [37]. Implementing public policies that enhance marriages and family care systems can decrease the likelihood of unhealthy lifestyles derived from loneliness.

Furthermore, a significant association was observed between reporting a poor health status and educational attainment. Logistic regression analysis revealed that individuals with higher education had a lower likelihood of reporting a poor health status compared to those with below primary school education. The statistical significance (1% probability) of the overall education-based concentration index highlights a concentration of poor health among less-educated individuals in the KSA. Although Brinda et al. [38] found no association between education and poor health status, other studies have confirmed this relationship [39,40]. This is expected as individuals with higher education tend to possess greater awareness about diseases and their impacts, along with better availability and access to quality health services, which explains their better health reporting. Advocating for policies that promote higher education levels in the population would contribute to addressing poor health.

Moreover, there was a significant association between reporting a poor health status and health insurance coverage status. The concentration of reporting a poor health status was higher among uninsured individuals (20.37%) compared to those with insurance coverage (9.46%). The likelihood of reporting a poor health status was also lower among those with health insurance compared to those without insurance in the logistic regression. Although Fonta et al. [28] found no significant difference in SRH between insured and uninsured older individuals, Kunna et al. [4] emphasized that the absence of health insurance coverage contributed to approximately 12% of observed health inequalities. Therefore, in addition to old age being a predisposing factor for poor health, access to health insurance is an important consideration for governments to enhance the health situation of the older population. The absence of insurance increases the likelihood of facing significant out-of-pocket expenses, which can result in catastrophic health expenditure among the aging population [41]. Similarly, there was a significant association between reporting a poor health status and chronic illness. Individuals suffering from chronic diseases had a higher likelihood of reporting a poor health status compared to those not suffering from chronic diseases, indicating that individuals with chronic conditions were approximately 4.1 times more likely to report a poor health status. This finding aligns with previous studies as the presence of a chronic illness places a burden on healthcare expenses and is often accompanied by the reporting of poor health status [42].

Lastly, the likelihood of reporting a poor health status was lower among higher income groups, except for those earning between 20,000 to 30,000 SR and those earning ≥ 30,000 SR, in comparison to the lower income group earning below 3000 SR. Similar findings were reported in China [43], Brazil [33,39], and Mexico [44]. Read et al. [45] found a significant association between self-reporting poor health, low income, and general poor socio-economic status among older individuals in Europe. Achdut and Sarid [46] argued that low income can hinder social participation and lead to poorer health outcomes for older adults. Melchiorre [47] highlighted the impact of worry, depression, and stress on the health of older individuals, particularly when facing financial constraints. A stable source of income during old age enhances social status, living conditions, and reduces worries. These findings underscore the importance of addressing income inequalities and providing financial support to improve the health and well-being of older adults.

This study possesses several notable strengths. The results are based on a comprehensive dataset that represents the entire nation, focusing exclusively on the health of the older population (≥60 years) of the KSA. Multiple techniques were employed to mitigate potential biases and enhance the validity of the findings and conclusions. As a result, the study holds relevance for informing policy design and implementation aimed at addressing health inequalities among the aging population. However, certain limitations are also associated with this study. Recall and individual biases are possible given the nature of the self-reported data. Future analyses could benefit from incorporating alternative data sources or employing standardized measures to minimize the impact of self-reporting. Additionally, due to its cross-sectional nature, the study cannot establish definitive causal relationships between the investigated factors and poor health.

## 5. Conclusions

Using the 2018 Saudi FHS and multiple analytical techniques, this study examined inequalities in self-reported health among older adults in the KSA and the factors that drive this inequality. The study highlights a high occurrence of self-reported poor health in individuals aged ≥70 years, particularly those grappling with chronic diseases. Moreover, there is a clustering of poor health among individuals with lower levels of education and those with limited income. The study has implications for research, policy, and practice. Firstly, concerning research implications, the study underscores the significance of examining inequality within specific segments of the population. This approach yields insights that may be overlooked when research focuses on general health inequalities. Furthermore, future studies could attempt to use panel data, observing adults over time, to progress toward establishing causal relationships. Secondly, in terms of policy and practice implications, the study highlights that some subgroups among the older population, especially those grappling with chronic diseases, face greater vulnerability and require targeted assistance. Therefore, the government needs to prioritize the implementation of social and health promotion programs that actively support these subpopulations of older adults in the KSA. To mitigate the health effects of aging, as indicated by this study, programs should focus on enhancing physical activity, promoting better nutrition, and addressing feelings of loneliness. By focusing on primary prevention measures, healthy aging can be promoted, ensuring the long-term sustainability of healthcare systems. Safeguarding the health of the older population will not only improve their well-being but also contribute to reduced government expenditure on healthcare funds.

## Figures and Tables

**Table 1:** Descriptive statistics of the sample (*n* = 2023).

Variable	Frequency	%
**Self-reported health**		
Good health	1672	82.65
Poor health	351	17.35
**Age group**		
Younger (60–69 years)	1104	54.57
Older (≥70 years)	919	45.43
**Gender**		
Woman	871	43.05
Man	1152	56.95
**Marital status**		
Unmarried	470	23.23
Married	1553	76.77
**Education level**		
Below primary school	1118	55.26
Primary school	307	15.18
Intermediate school	236	11.67
Secondary school	226	11.17
Higher education	136	6.72
**Nationality**		
Non-Saudi	191	9.44
Saudi	1832	90.56
**Monthly income (Saudi Riyal)**		
˂3000	168	8.30
3000 to <5000	349	17.25
5000 to <7000	310	15.32
7000 to <10,000	416	20.56
10,000 to <15,000	363	17.94
15,000 to <20,000	142	7.02
20,000 to <30,000	107	5.29
≥30,000	168	8.30
**Insurance status**		
Not insured	1463	72.32
Insured	560	27.68
**Suffering from chronic illness**		
No	285	14.09
Yes	1738	85.91

**Table 2:** Bivariate analysis of self-reported health and socio-economic characteristics (*n* = 2023).

Variable	Frequency	Percent of Self-Reported Poor Health	Chi-Square
**Age group**			46.05 ***
Younger (60–69 years)	1104	12.14	
Older (≥70 years)	919	23.61	
**Gender**			32.23 ***
Woman	871	22.85	
Man	1152	13.19	
**Marital status**			51.17 ***
Unmarried	470	28.51	
Married	1553	13.97	
**Education level**			78.72 ***
Below primary school	1118	23.43	
Primary school	307	11.73	
Intermediate school	236	5.51	
Secondary school	226	15.93	
Higher education	136	2.94	
**Nationality**			8.06 ***
Non-Saudi	191	9.95	
Saudi	1832	18.12	
**Monthly income (Saudi Riyal)**			80.67 ***
˂3000	168	30.36	
3000 to <5000	349	14.33	
5000 to <7000	310	23.23	
7000 to <10,000	416	13.46	
10,000 to <15,000	363	11.02	
15,000 to <20,000	142	4.93	
20,000 to <30,000	107	31.78	
≥30,000	168	24.4	
**Insurance status**			33.58 ***
Not insured	1463	20.37	
Insured	560	9.46	
**Suffering from chronic illness**			35.79 ***
No	285	4.91	
Yes	1738	19.39	

*** *p* < 0.01.

**Table 3:** Wagstaff inequality indices for self-reported health by income and education level (*n* = 2023).

Variable	Income		Education	
	Index Estimate	95% CI	Index Estimate	95% CI
**National level**	−0.061	(−0.126 to 0.005)	−0.261 ***	(−0.320 to −0.202)
**Age group**				
Younger (60–69 years)	0.358 ***	(0.257 to 0.459)	−0.166 ***	(−0.263 to −0.068)
Older (≥70 years)	−0.288 ***	(−0.373 to −0.203)	−0.280 ***	(−0.353 to −0.207)
**Gender**				
Woman	−0.001	(−0.100 to 0.081)	−0.213 ***	(−0.278 to −0.149)
Man	−0.125 **	(−0.223 to −0.028)	−0.173 ***	(−0.285 to −0.191)
**Marital status**				
Unmarried	−0.067	(−0.181 to 0.047)	−0.171 ***	(−0.244 to −0.099)
Married	0.131	(−0.069 to 0.095)	−0.222 ***	(−0.299 to −0.145)
**Nationality**				
Non-Saudi	−0.807 ***	(−1.054 to −0.560)	−0.620 ***	(−0.870 to −0.369)
Saudi	−0.028	(−0.095 to 0.040)	−0.221 ***	(−0.282 to −0.161)
**Insurance status**				
Not insured	0.133 ***	(0.061 to 0.205)	−0.185 ***	(−0.245 to −0.123)
Insured	−0.743 ***	(−0.893 to −0.593)	−0.321 ***	(−0.478 to −0.163)
**Suffering from chronic illness**				
No	−0.424 ***	(−0.727 to −0.120)	−0.260 *	(−0.550 to 0.030)
Yes	−0.056 *	(−0.124 to 0.011)	−0.256 ***	(−0.317 to −0.195)

*** *p* < 0.01, ** *p* < 0.05, * *p* < 0.1.

**Table 4:** Association between poor health status and socio-economic factors (logistic regression).

Variable	Model 1		Model 2		Model 3	
	OR	95% CI	OR	95% CI	OR	95% CI
**Age group**						
Younger (60–69 years)	Reference		Reference		Reference	
Older (≥70 years)	2.075 ***	(1.589–2.709)	1.797 ***	(1.387–2.328)	2.035 ***	(1.547–2.675)
**Gender**						
Woman	Reference		Reference		Reference	
Man	0.720 **	(0.526–0.986)	0.728 *	(0.524–1.011)	0.896	(0.629–1.276)
**Marital status**						
Unmarried	Reference		Reference		Reference	
Married	0.584 ***	(0.409–0.835)	0.673 **	(0.486–0.933)	0.566 ***	(0.393–0.815)
**Education level**						
Below primary school			Reference		Reference	
Primary school			0.640 **	(0.430–0.952)	0.616 **	(0.406–0.934)
Intermediate school			0.282 ***	(0.155–0.510)	0.242 ***	(0.129–0.453)
Secondary school			1.227	(0.784–1.919)	1.001	(0.618–1.618)
Higher education			0.225 ***	(0.078–0.645)	0.181 ***	(0.061–0.535)
**Nationality**						
Non-Saudi	Reference		Reference		Reference	
Saudi	1.676 *	(0.958–2.933)	1.235	(0.711–2.145)	1.302	(0.733–2.312)
**Monthly income**						
˂3000	Reference				Reference	
3000 to <5000	0.392 ***	(0.241–0.636)			0.384 ***	(0.236–0.624)
5000 to <7000	0.881	(0.549–1.413)			0.913	(0.567–1.470)
7000 to <10,000	0.387 ***	(0.241–0.624)			0.446 ***	(0.275–0.725)
10,000 to <15,000	0.382 ***	(0.231–0.631)			0.439 ***	(0.262–0.735)
15,000 to <20,000	0.247 ***	(0.104–0.586)			0.397 **	(0.163–0.968)
20,000 to <30,000	1.656 *	(0.932–2.942)			2.120 **	(1.160–3.876)
≥30,000	1.350	(0.769–2.369)			1.751 *	(0.967–3.171)
**Insurance status**						
Not insured	Reference		Reference		Reference	
Insured	0.410 ***	(0.290–0.581)	0.521 ***	(0.369–0.734)	0.480 ***	(0.335–0.687)
**Suffering from chronic illness**						
No	Reference		Reference		Reference	
Yes	4.069 ***	(2.302–7.193)	4.235 ***	(2.417–7.418)	4.089 ***	(2.305–7.251)

Abbreviations: CI, confidence interval; OR, odds ratio. *** *p* < 0.01, ** *p* < 0.05, * *p* < 0.1.

## Data Availability

The datasets generated and/or analyzed during the current study are not publicly available due to privacy, confidentiality, and other restrictions. Access to data can be gained through the General Authority for Statistics in Saudi Arabia via https://www.stats.gov.sa/en.
